# Health assessment of wild speckled dwarf tortoises, *CHERSOBIUS SIGNATUS*

**DOI:** 10.1186/s12917-021-02800-5

**Published:** 2021-03-04

**Authors:** Livio Galosi, Anna Rita Attili, Stefania Perrucci, Francesco C. Origgi, Adolfo Maria Tambella, Giacomo Rossi, Vincenzo Cuteri, Maira Napoleoni, Nicholas Aconiti Mandolini, Gianni Perugini, Victor J. T. Loehr

**Affiliations:** 1 School of Biosciences and Veterinary Medicine, University of Camerino, 62024 Matelica, Italy; 2 Department of Veterinary Science, University of Pisa, 56126 Pisa, Italy; 3 Centre for Fish and Wildlife Health (FIWI), DIP, Vetsuisse Faculty, University of Bern, 3001 Bern, Switzerland; 4 Experimental Zooprophylactic Institute (IZS) of Umbria and Marche ‘Togo Rosati’, 06126 Perugia, Italy; 5 Dwarf Tortoise Conservation, Kwikstaartpad 1, 3403ZH IJsselstein, Netherlands

**Keywords:** Health assessment, *Chersobius [Homopus] signatus*, Reptile, Tortoise, Wildlife

## Abstract

**Background:**

In free-ranging reptile populations, bacterial, fungal, viral and parasitic pathogens may affect hosts through impairment in movements, thermoregulation, reproduction, survival, and population dynamics. The speckled dwarf tortoise (*Chersobius* [*Homopus*] *signatus*) is a threatened species that is mostly restricted to the Succulent Karoo biome in South Africa, and little information on pathogens of this species is available yet. We derived baseline parameters for five males and five females that were captured to genetically enhance a conservation breeding program in Europe. Upon collection of the tortoises, ticks were removed and identified. Immediately upon arrival in Europe, ocular, nasal, oral and cloacal swabs were taken for viral, bacteriological and mycological examinations. Fecal samples were collected before and 1 month after fenbendazole treatment, and analyzed for parasites. A panel of PCR, aiming to detect herpesviruses, adenoviruses and iridoviruses, was carried out.

**Results:**

Samples were negative for viruses, while bacteriological examination yielded detectable growth in 82.5% of the swabs with a mean load of 16 × 10^7^ ± 61 × 10^8^ colony forming units (CFU) per swab, representing 34 bacterial species. Cloacal and oral swabs yielded higher detectable growth loads than nasal and ocular swabs, but no differences between sexes were observed. Fungi and yeasts (mean load 5 × 10^3^ ± 13 × 10^3^ CFU/swab) were detected in 25% of the swabs. All pre-treatment fecal samples were positive for oxyurid eggs, ranging from 200 to 2400 eggs per gram of feces, whereas after the treatment a significantly reduced egg count (90–100% reduction) was found in seven out of 10 individuals. One remaining individual showed 29% reduction, and two others had increased egg counts. In five tortoises, *Nycthocterus* spp. and coccidian oocysts were also identified. Soft ticks were identified as *Ornithodoros savignyi*.

**Conclusions:**

Our baseline data from clinically healthy individuals will help future studies to interpret prevalences of microorganisms in speckled dwarf tortoise populations. The study population did not appear immediately threatened by current parasite presence.

## Background

Free-ranging reptiles may be infected by bacteria, fungi, viruses and parasites, which can affect hosts through impairment of movements, thermoregulation, reproduction, survival, and population dynamics [1–3]. Accordingly, pathogens can represent challenges for wildlife conservation, particularly when acting in conjunction with anthropogenic stressors [4, 5]. Assessing the composition and epidemiology of pathogen communities in wild host populations is, therefore, essential for the development of host conservation management.

Tortoise populations are declining world-wide, leading the IUCN [6] to categorize 65% of all extant species [7] threatened with extinction (i.e., categories Vulnerable and [Critically] Endangered). Primary causes for decline are habitat destruction, harvest for food, use of body parts for traditional medicine and pet trade, but some tortoise populations are also under threat of infections by some virulent pathogens [2]. Relatively few wild populations have been rigorously assessed yet.

The speckled dwarf tortoise (*Chersobius* [*Homopus*] *signatus*) is mostly restricted to a small range in the Succulent Karoo biome in South Africa, where its numbers are declining [8, 9]. The decline is primarily caused by habitat destruction and degradation, climate change and increased predation [9], but other factors may exacerbate their effects. About speckled dwarf tortoises, little information is available on infestations by mutual and commensal organisms. One study suggested that ticks might switch from mammalian hosts to speckled dwarf tortoises during drought [10], potentially increasing the impact of drought on the tortoises. Given the lack of knowledge concerning if and how pathogens may contribute to the decline of speckled dwarf tortoises, the aims of this study were: 1) to derive baseline data on mutual, commensal and pathogenic organisms found in *C. signatus*; 2) to investigate their distribution among sexes and anatomical district; 3) to assess if their presence may pose a threat to the sampled *C. signatus* population.

## Results

### Bacteriological and mycological examinations

All tortoises harbored bacterial species. Most of the collected swabs (82.5%, *n* = 40) yielded bacterial colonies with a significant difference observed among anatomical districts (χ^2^ = 11.60, *P* = 0.011, d.f. = 3). The anatomical districts most colonized were the cloacal (100%, *n* = 10) and the oral cavities (100%, *n* = 10), with less frequent colonization of the nasal cavity (50%, *n* = 10) and conjunctival sacs (80%, *n* = 10). The mean of total bacterial loads was 16 × 10^7^ ± 61 × 10^8^ CFU/swab. Cloacal (244 × 10^7^ ± 794 × 10^7^ CFU/swab), and oral cavities (158 × 10^5^ ± 452 × 10^5^ CFU/swab) yielded higher counts than conjunctival sacs (13 × 10^3^ ± 25 × 10^3^ CFU/swab) and nasal cavity (33 × 10^4^ ± 51 × 10^4^ CFU/swab), but differences among anatomical districts were not significant (*F* = 1.76, *P* = 0.173. d.f. = 36).

Overall, thirty-four different bacterial species were isolated (Table 1). *Bacillus cereus* (13 isolates), *Mycoplasma* spp. (9 isolates), *Salmonella enterica subsp. salamae* serovar 6,7:a:z42 (13 isolates) were commonly detected, especially in the cloacal and oral swabs. *Aeromonas* spp., *Enterobacter sakazakii*, *Yersinia* spp., *Vibrio* spp., *Campylobacter* spp., *Helicobacter* spp. lacked in all isolates. Gram positive represented the most frequent bacteria (49.6% of all positive isolates), followed by Gram negative bacteria (38.7%), *Mycoplasma* spp. (7.6%), and *Ureaplasma* spp. (4.2%) (χ^2^ = 72.70, *P* < 0.001, d.f. = 3).

**Table 1:** Frequencies of bacterial species and isolations from different anatomical districts in five male and five female speckled dwarf tortoises (*Chersobius* [*Homopus*] *signatus*). Absence of a frequency value indicates that the bacterial species was not isolated. Two ocular and five nasal swabs tested negatively

Bacterial species	Eye (*n* = 10)	Nose (*n* = 10)	Mouth (*n* = 10)	Cloaca (*n* = 10)
*Acinetobacter calcoaceticus*	1		1	
*Aerococcus* spp.				2
*Alcaligenes faecalis*			1	
*Bacillus cereus*	2		3	8
*Bacillus licheniformis*			1	
*Bacillus* spp.	3		3	3
*Bacillus thuringiensis*				1
*Brevibacterium* GpB	1			
*Brevundimonas diminuta*			2	1
*Burkholderia cepacia* complex	1	1		2
*Cedecea davisae*			1	
*Citrobacter freundii*				3
*Clostridium* spp.		1		
*Corynebacterium jeikeium*	1	1	1	1
*Enterococcus avium*				4
*Enterococcus faecalis*				4
*Escherichia coli*				1
*Hafnia alvei*			1	
*Kytococcus aerolatus*	1			
*Kytococcus schroeteris*	2			1
*Listeria* spp.				1
*Micrococcus luteus*		1	1	
*Mycoplasma* spp.		1	2	6
*Neisseria weaveri /elongata*			1	
*Pantoea agglomerans*			1	4
*Pseudomonas fluorescens/putida*			3	2
*Salmonella subsp. salamae 6,7:a:z42*	1	1	2	9
*Shigella* spp.			1	1
*Staphylococcus auricularis*	1		2	
*Stenotrophomonas maltophilia*			2	2
*Streptococcus equinus*			1	3
*Streptococcus intermedius*				1
*Streptococcus mutans*			2	2
*Ureaplasma* spp.		1	1	3
**Total species**	**10**	**7**	**21**	**23**
**Total bacterial isolations**	**14**	**7**	**33**	**65**

**Fig. 1:**
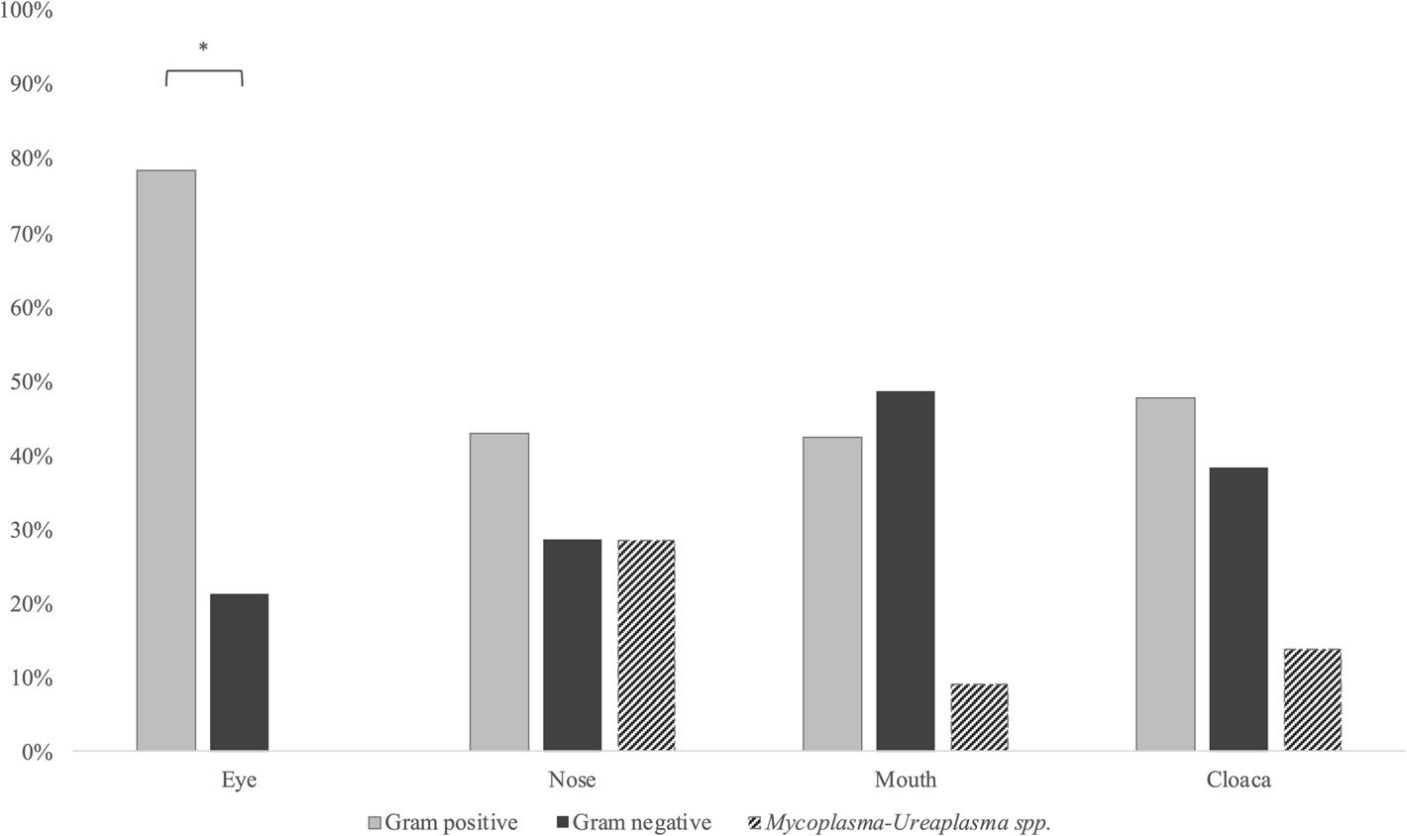
Bacterial frequencies (%) observed in five male and five female speckled dwarf tortoises (*Chersobius* [*Homopus*] *signatus*) in relation to anatomical district and Gram affinity. [*: χ^2^ = 8.74, *P* = 0.017]

When *Mycoplasma* spp. and *Ureaplasma* spp. were combined, the frequencies of Gram positive, Gram negative, and *Mycoplasma*-*Ureaplasma* spp. isolates differed significantly among anatomical districts (χ^2^ = 35.032, *P* < 0.001, d.f. = 6; Fig. 1). More specifically, a difference between Gram positive and negative was observed in ocular swabs, (78.6% vs. 21.4%), where *Mycoplasma* spp. and *Ureaplasma* spp. were absent (Fig. 1). *Mycoplasma* spp. and *Ureaplasma* spp. accounted for a mean load of 504 × 10^3^ ± 515 × 10^3^ CFU/swab. They were cultured in nasal (28.6%, 1 × 10^6^ ± 0 CFU/swab), cloacal (13.8%, 561 × 10^3^ ± 520 × 10^3^ CFU/swab) and oral swabs (9.1%, 1 × 10^3^ ± 0 CFU/swab). No significant differences were recorded among anatomical districts, neither for prevalence (χ^2^ = 3.11, *P* = 0.795, d.f. = 3) nor for the average loads recorded (*F* = 3.25, *P =* 0.078, d.f. = 36).

**Fig. 2:**
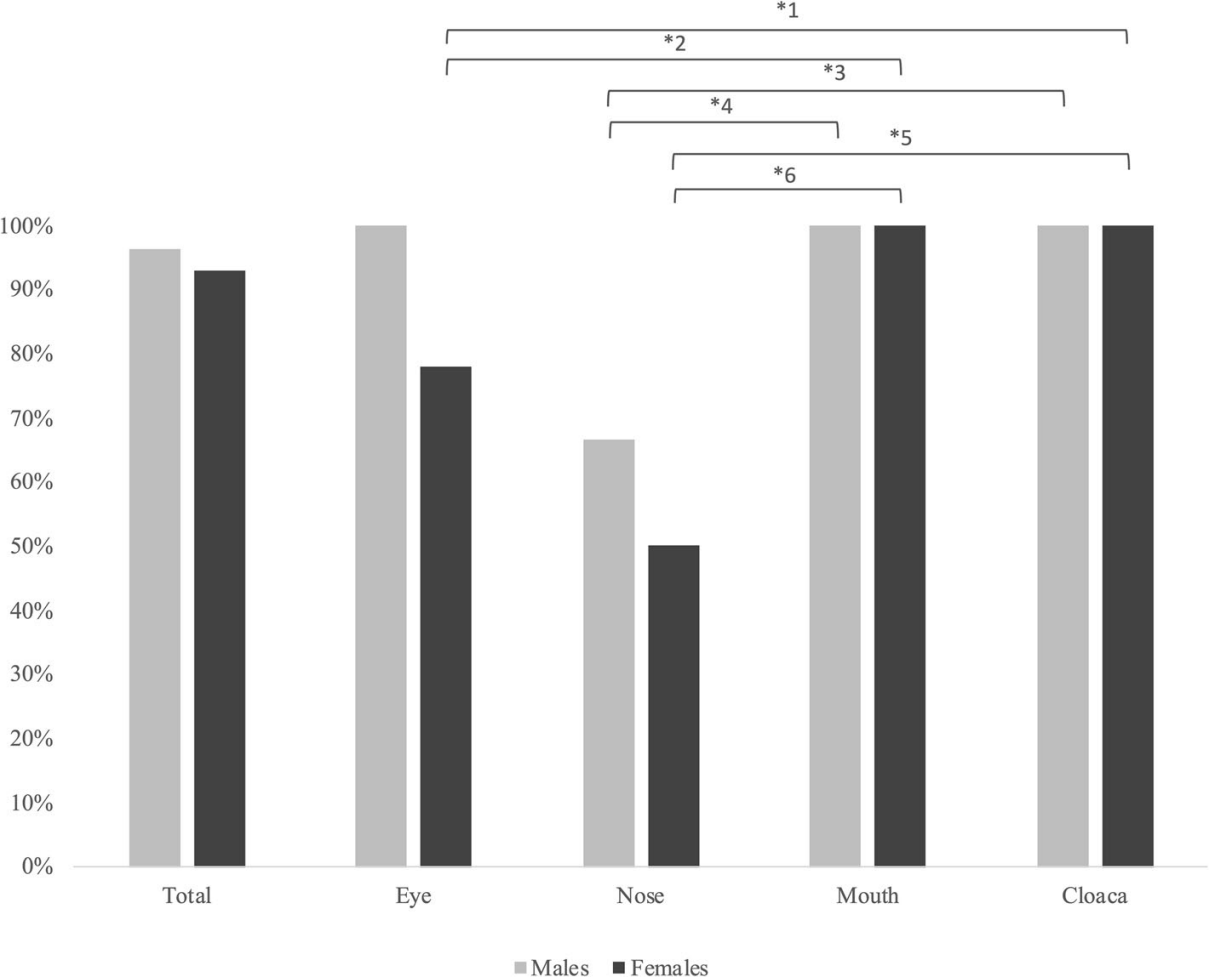
Bacterial frequencies (%) observed in five male and five female speckled dwarf tortoises (*Chersobius* [*Homopus*] *signatus*) in relation to sex and anatomical district. [^*1^: χ^2^ = 4.37, *P* = 0.034; ^*2^: χ^2^ = 8.60 *P* = 0.003; ^*3^: χ^2^ = 9.92, *P* = 0.002; ^*4^: χ^2^ = 5.53, *P* = 0.019; ^*5^: χ^2^ = 19.89, *P* < 10^4^; ^*6^: χ^2^ = 10.29, *P* = 0.001]

The frequencies of Gram positive and Gram negative bacterial isolates in males and females were similar (χ^2^ = 0.64, *P* = 0.42, d.f. = 1). However, analysis performed within each gender sub-group showed different frequencies of isolates among anatomical districts (χ^2^ = 68.31, *P* < 0.001. d.f. = 3; Fig. 2), with the largest frequencies in cloacal and oral swabs. Both sexes had a similar mean bacterial load (150 × 10^7^ ± 697 × 10^7^ and 487 × 10^6^ ± 217 × 10^7^ CFU/swab for males and females, respectively, *t* = − 0.663, *P* = 0.511, d.f. = 38), with large individual variation within males.

Yeasts and fungi were observed in 25% of swabs with a mean fungal load of 3.5 × 10^3^ ± 13 × 10^3^ CFU/swab. Yeasts represented 78.6% of positive swabs (mean load 7 × 10^3^ ± 14 × 10^3^ CFU/swab), and fungi 21.4% (20 ± 10 CFU/swab; *t* = 0.77, *P* = 0.456). *Rhodotorula rubra*, *R. mucilaginosa*, *Candida* spp. and *Alternaria* spp. were over-represented in ocular and oral swabs (Table 2). In ocular swabs, yeasts resulted 13 × 10^3^ ± 20 × 10^3^ CFU/swab (*n* = 5), in cloaca 2 × 10^3^ ± 1 × 10^3^ CFU/swab (*n* = 3), and in mouth 20 ± 17 CFU/swab (*n* = 3). *Penicillium* spp. was identified only in nasal swabs.

**Table 2:** Frequency/swab of fungal species isolated from different anatomical districts in five male and five female speckled dwarf tortoises (*Chersobius* [*Homopus*] *signatus*)

Fungal species	Eye (*n* = 10)	Nose (*n* = 10)	Mouth (*n* = 10)	Cloaca (*n* = 10)
*Alternaria* spp.	1			
*Candida* spp.			1	1
*Cryptococcus laurentii*	1			
*Fusarium* spp.			1	
*Geotrichum* spp.				1
*Penicillum* spp.		1		
*Rhodotorula mucilaginosa*	1		1	
*Rhodotorula rubra*	3			1
*Trichophyton terrestre*			1	
**Total fungal isolations**	**6**	**1**	**4**	**3**
Negative	5	9	7	9

### Virological examination

None of the samples revealed the presence of detectable amounts of herpesvirus, iridovirus or adenovirus DNA.

### Parasitological examination

**Fig. 3:**
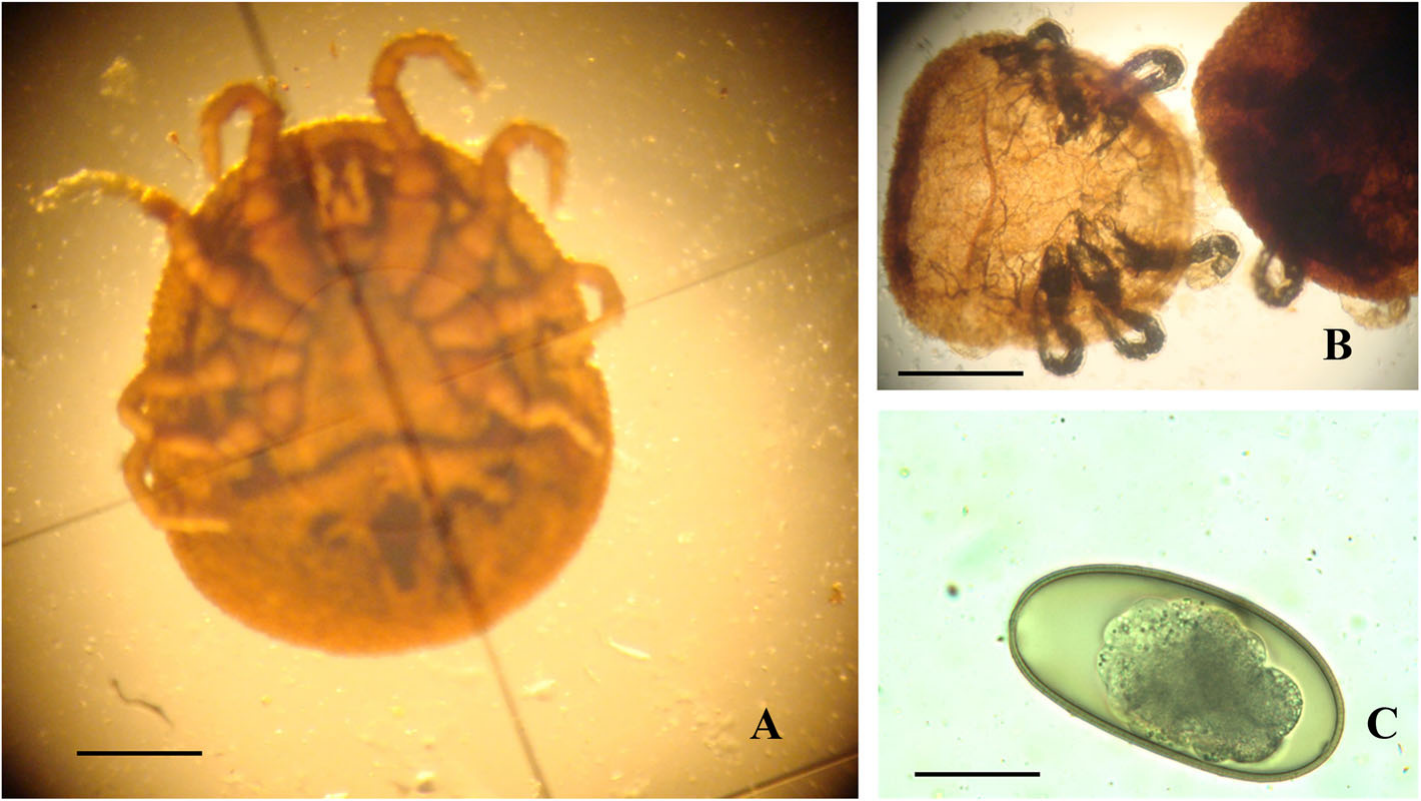
Parasites identified in 10 speckled dwarf tortoises (*Chersobius* [*Homopus*] *signatus*). **a** *Ornithodoros savignyi* nymph (scale bar 0.45 mm); **b**) *Ornithodoros savignyi* larvae (scale bar 0.4 mm); **c**) Oxyurid egg (scale bar 35 μm)

All tortoises were infested by morphologically similar ticks, although at different stages of development. Based on morphology, number of limbs and absence of the typical shield of the hard ticks, they were identified as larvae and nymphs of soft ticks. Light brown nymphal stages (Fig. 3a) had an oval body, with a wider posterior gently rounded to form a sub-triangular shape and four-paired legs of moderate length and slender. They measured about 0.988–2.457 mm in length and 0.936–2.340 mm in width. Larvae have a semicircular and dark brown body and three pairs of legs (Fig. 3b). They are 1.10 mm in length and 1.0 mm in width. Morphological and metric characteristics of collected ticks permitted their identification as *Ornithodoros savigny*.

All fecal samples collected before the treatment were positive for oxyurid eggs (Fig. 3c) with a FEC ranging from 200 to 2400 EPG. Egg dimensions ranged from 83 to 125 μm in length and 52–70 μm in width. A FECR higher than 90% was observed in 7 out of 10 treated animals (Table 3). Moreover, few *Nycthocterus* spp. ovoid cysts of about 50 μm in length and trophozoites, about 30–40 μm in length, and subspherical coccidian oocysts of about 20–23.4 × 17–23.4 μm were identified in five tortoises at flotation test, before and after the fenbendazole treatment.

**Table 3:** Fecal egg count reduction (FECR) of pinworm eggs observed in 10 wild speckled dwarf tortoise (*Chersobius* [*Homopus*] *signatus*) fecal samples prior and one month after deworming with Fenbendazole (75 mg/kg, repeated with 50 mg/kg after 10 days)

Animal	FEC before the treatment (EPG)	FEC after the treatment (EPG)	FECR
1	2400	Negative	100%
2	< 50	Negative	100%
3	1000	Negative	100%
4	< 50	100	No reduction
5	< 50	Negative	100%
6	350	Negative	100%
7	1400	100	93%
8	350	Negative	100%
9	700	500	29%
10	200	800	No reduction

## Discussion

This study provides the first comprehensive analysis of microorganisms and parasites in free-ranging speckled dwarf tortoises, and expands previous recordings of nematodes [11] and ticks [10] in the same species.

All sampled individuals appeared clinically healthy, suggesting that encountered microorganisms might be commensal and accordingly, not posing an immediate threat to the population in absence of specific stressors. “Commensalism” is defined as a state of infection that results in either no damage or clinically inapparent damage to the host, though it can elicit an immune response [12]. Evolutionary co-existence has equipped both the micro-organisms and the immune system of the host with similar mechanisms of defense and selection [13], however the interaction with microbes can be harmful for the host and opportunistic infections can occur [14]. These definitions and these scenarios, are becoming more complex at the light of the role of the environment as a critical third player, when it comes to host-pathogen interaction in free ranging individuals. It is becoming more and more evident that the delicate balance and equilibrium existing between host and the components of its microbiological community may be challenged and subverted by environmental factors, which may impact the host immune response in many ways, from the availability of critical resources as food to, in case of poikilotherms, as tortoises are, anything that could influence the environmental temperature and regularity of seasonality [15]. Accordingly, the significance of the identified organisms, within the disease ecology of *C. signatus*, needs to be evaluated also at the light of the possible role that emerging environmental stressors might have and on the information that will be collected in follow up studies.

The ongoing “-omic” revolution, provides now very powerful investigation tools, which allow a very complete and thorough characterization of very complex microbiological communities present in specific hosts. Our investigation was based on conventional culture-based, approach, which although might appear reductive at the light of the currently available technology, was considered ideal for an initial screening of the *C. signatus* microbiological environment and functional to the isolation of viable organisms, which might be critical for future in vitro and in vivo, and follow up studies, which we plan to enrich as well with a Next Generation Sequencing (NGS) approach.

Along with the considerations made above, even when considered part of the natural microbiota of the tortoises, some bacterial species may have clinical significance, mostly as opportunistic pathogens, both for animals and humans. Mycoplasma are frequently found in chelonians, where are often cause of severe disease [16, 17], but not are systematically associated with obvious pathology [18]. We found the presence of *Mycoplasma* spp. in four out of 10 examined animals. Our results showed a prevalence higher than that observed in a previous research [19], that reported a prevalence of 15.8% for *Mycoplasma agassizii* in captive tortoises (*Testudo* spp.) in the United Kingdom. In Italy, in particular on the Sardinia island, a similar prevalence of *Mycoplasma* spp. (37%) was found in tortoises belonging to the genus *Testudo* [20].

A clear predominance of Gram-positive species can be noticed, in accordance with data reported for Geoffroy’s side-necked turtle (*Phrynops geoffranus*) [21], in particular in the conjunctival flora. Only one species of *S. auricularis* was recorded in this anatomical district and it could be considered part of saprophyte microflora, although in few studies this species has been considered responsible of ocular lesions in some reptiles [22, 23]. To the best of our knowledge, this study is the first report of *Kytococcus areolatus* and *Kytococcus schroeteri* in chelonians. *K. aerolatus* was first identified from an indoor air sample and has never been reported to cause human infections [24], whereas *K. schroeteri* was identified in bacteraemia in a human patient [25]. A strain of *Listeria* spp. was also detected, but it was not identified as *L. monocytogenes*. However, this bacterial genus was reported as nonpathogenic in European pond turtles (*Emys orbicularis*) [26, 27].

Several studies analyzed microorganisms in free ranging wild reptiles and investigated different anatomical sites. In Brazil, the Pantanal alligator (*Caiman crocodilus yacare*) and the “jacarétinga” (*Caiman crocodilus crocodilus*) carried *Aeromonas* spp*.*, *Acinetobacter* spp*.*, *Citrobacter freundii, Escherichia coli* and *Pseudomonas* spp. [28], while *Salmonella* spp. was found in 39.1% of pet reptiles, including chelonians [29]. In the same country, *Escherichia coli, Klebsiella pneumoniae, Enterobacter agglomerans, Citrobacter freundii* and *Bacillus* spp. were identified in oral samples of Geoffroy’s side-necked turtles [21].

Although disease associated with Gram-positive bacteria is sporadic, Gram-negative bacteria are commonly reported as pathogens of reptiles [30, 31]. Different Gram-negative bacteria were identified in this study. *Acinetobacter calcoaceticus*, detected in oral and conjunctival swabs for *C. signatus*, has previously been reported in the oral cavity of *Caretta caretta* in poor general conditions [32], and in nasal swabs from *Testudo graeca* showing rhinitis [33]. *Citrobacter* spp. has been identified as the main microorganism associated with septicemic cutaneous ulcerative disease (SCUD) or other necrotic or ulcerative changes [34]. Nevertheless, the three *C. signatus* found positive for *Citrobacter freundii* in this study were asymptomatic*. Stenotrophomonas maltophilia* may cause several opportunistic infections as sepsis, pneumonia, urinary tract infection, meningitis, endocarditis, septic arthritis, and peritonitis in humans [35]. This bacterium was isolated from Red-footed tortoise (*Geochelone carbonaria*) with a respiratory infection [36] and in captive snakes with oral bacterial infection [37], but in this study it was isolated from oral and cloacal cavities of two healthy tortoises and not associated to detectable lesions.

*E. coli* and *P. aeruginosa,* reported as the prevalent bacterial species in several previous studies [27, 38–40], were not detected in this study.

It is well known that chelonians can carry bacteria with zoonotic potential [29, 41, 42], mainly *Salmonella* spp., that is prevalent in high rates in both captive and free-ranging turtles [43]. In our study, the prevalence of *Salmonella* spp. was relatively low (10.9%). This result is similar to data reported in exotic and native turtles in Spain [44], and in Polish European pond turtle (*Emys orbicularis*) [27]. In contrast, 100% incidence of *Salmonella* was recorded in free-ranging Spur-thighed tortoises (*Testudo graeca*) living in a National Park in southwestern Spain [45]. The ingestion of feces or contaminated water is considered a probable way of transmission [46].

In previous studies, several bacteria including *Burkholderia cepacia* were found associated with exudative bronchopneumonia and/or granulomatous pneumonia and other bronchopneumonic lesions of marine turtles [32, 47, 48]. In our investigation *Burkholderia cepacia* complex (Bcc) was identified in two tortoises, in one female from conjunctival, nasal and cloacal swabs, and in one male only from cloacal cavity. Bcc is a group of Gram negative opportunistic pathogens that can be found in soil and water [49, 50], in healthy fishes [51], but it has also been detected in humans with necrotizing pneumonia [52].

Usually, reptiles colonized with yeasts do not show any clinical sign, as in our case. *Rhodotorula* spp. are considered emerging opportunistic pathogens in humans [53]. *R. mucilaginosa* is a species frequently found in plastrons and, together with *Cryptococcus laurentii*, in oral cavities of two chelonian species in Brazil [54]. Most of the soil yeasts and fungi, belonging to Hyalohyphomycetes (e.g. *Fusarium*) and Zygomycetes (e.g. *Alternaria*), are implicated in infections in human patients, ranging from colonization and localized infections in immunocompetent individuals to fungemia and disseminated diseases in immunocompromised patients [55].

Oxyurids (pinworms) are generally the most frequent internal nematodes identified in tortoises. These nematodes, localized in the large intestine, are generally considered to be almost commensal [56]. The low pathogenicity [57] and the monoxenous life cycle [58] of these parasites are considered the main reasons for their large distribution among tortoises. However, in heavily infested tortoises, pinworms may be a potential cause of intestinal obstruction, anorexia and death after hibernation [56]. Oxyurid infection showed a prevalence of 100% in the tortoises examined here. Thirty days after the treatment with fenbendazole, 100% efficacy was observed in seven out of ten treated animals. However, four tortoises remained positive and in two animals the number of eggs was higher than it was prior the treatment. This result could depend on incomplete efficacy of the therapeutic protocol used, but it cannot be completely excluded that this result may also depend on a new infection. In any case, although in some previous studies the therapeutic protocol used has been proven to be effective for the treatment of pinworms of other turtle species [59] and despite fenbendazole is commonly used in veterinary medicine [60], in Hermann’s tortoises (*Testudo hermanni*) it can cause hematological and biochemical changes that may indicate transient immune suppression [61]. As our results suggest that the protocol used for the treatment of oxyurids was not totally effective and the clinicopathological changes reported as possible side effects [61] were not examined in this study, further investigations are needed to establish the risk/benefit ratio in oxyurid treatment in speckled dwarf tortoises.

*Nycthocterus* spp. are ciliated protozoans of the chelonian digestive-tract flora that may play an important role in the digestion of cellulose. Therefore, in most cases, these protozoan cause little or no damage to reptiles [62]. However, in heavy infections or in stress conditions *Nycthocterus* infections may cause severe irritation of the intestinal wall and colitis, diarrhoea, dehydration, weight loss and passage of undigested food in the feces have been recently reported in infected captive turtles [63].

Eimeriid coccidia commonly infect turtles and might contribute to morbidity and mortality under captive conditions. These protozoan parasites typically show tissue specificity, usually being limited to the epithelium of the gut. However, disseminated infections have been reported in vertebrates (i.e. birds and mammals) as in turtles [64, 65]. *Eimeria* spp. are the most common coccidia species in chelonians. However, the morphological identification of coccidian oocysts found in positive *C. signatus* was not possible because this would require live parasites.

This study is the first report of protozoan infections, i.e. *Nycthocterus* spp. and coccidia, in the speckled dwarf tortoise. The tick species that we identified, *O. savigny*, is distributed throughout Africa and has already been reported in *C. signatus*, mainly on the hind limbs, forelimbs and neck of infested tortoises [10].

## Conclusions

Speckled dwarf tortoise populations are undergoing a dramatic decline due to habitat destruction, climate change and other threats [9]. The deteriorating conservation status of the taxon requires a pro-active approach to assess and understand emerging threats before they exacerbate threats to populations. We showed that speckled dwarf tortoise populations may host a wide range of microorganisms and metazoan species. Although sampled individuals appeared clinically healthy despite their bacterial and fungal loads, other stressors such as drought [10, 66] may induce shifts in parasites that could exacerbate negative effects in speckled dwarf tortoise populations [67].

This study identified no immediate conservation threats associated with microorganism infestations in examined speckled dwarf tortoises. Because changes in parasite populations, through stressors enacting on parasites or tortoises, might induce new threats, we recommend that the prevalence of actual and potential pathogenic species hosted by speckled dwarf tortoises should be considered when developing conservation measures for the taxon. Despite the small sample size is a limitation of this study, this health assessment provided very initial preliminary baseline data that we consider critical for future similar studies in this and other tortoise species. Further studies will be necessary to collect samples from *C. signatus* individuals in other locations and/or at different times of year, contributing to provide a more complete health assessment of the *C. signatus* free-ranging tortoise population and further understand the disease ecology of this species.

## Methods

### Animals

Five adult male and five adult female speckled dwarf tortoises (*Chersobius* [*Homopus*] *signatus*) were included in the study. The tortoises were collected between 11 and 19 September 2015 near Springbok, South Africa (permits FAUNA 053/2015, CITES 148487 and 15NL226435/11), to genetically enhance a European conservation breeding program. Ticks were immediately removed from each specimen at collection, and preserved in alcohol for further identification. Fenbendazole (Panacur, MSD Animal Health S.r.l., USA) was administered preventively 75 mg/kg, repeated with 50 mg/kg after 10 days, because of the large number of nematodes that had previously been observed in wild *C. signatus* [11]. Before and 1 month after the treatment [59], individual fecal samples (*n* = 10) were collected and preserved in 70% alcohol to be later examined for parasites. Immediately after capture, tortoises were transported in individual and isolated containers in order to prevent cross-contamination of pathogens among individuals. Samples for bacteriological, mycological and virological examinations were collected after the arrival in Europe to minimize the stress related to handling in the field conditions. Tortoises were held for a maximum of 10 days in the dark, at room temperature to reduce metabolic activity and stress related to handling and transport. After the study, the tortoises are maintained in captivity in Europe, for a conservation breeding program managed by the Dwarf Tortoise Conservation.

### Bacteriological and mycological examinations

Upon arrival in Europe, swabs from conjunctival sacs (*n* = 10), nasal districts (*n* = 10), oral cavities (*n* = 10) and cloacas (*n* = 10) were taken for bacteriological and mycological examinations. Swabs were immediately shipped to the laboratory on dry ice, and cultural examinations started as soon as the samples arrived at destination. Standard diagnostic protocols [68] and selective culture media were used for the detection of bacteria, including *Salmonella* spp., *Enterobacter sakazakii*, *Aeromonas* spp., *Listeria* spp., *Mycoplasma* spp., *Ureaplasma* spp., *Burkholderia cepacia* complex, *Yersinia* spp., *Vibrio* spp., *Campylobacter* spp., *Helicobacter* spp., *Bacillus cereus*, and *Clostridium* spp.

To evaluate the total fungal and bacterial loads, each swab was filled with 1 ml of sterile saline solution (ThermoFisher Oxoid, Italy) for 30 m at room temperature and then vortexed for 30 s. Aliquots of 100 μl were spread onto Sabouraud Dextrose Agar with chloramphenicol (Liofilchem®, Italy) and Mycosel Agar (Liofilchem®, Italy), for 7 d incubation at 30 °C, and on Plate Count Agar (Liofilchem®, Italy) for bacterial count after incubation at 37 °C for 24–72 h.

For qualitative bacteriological investigations, the extracted swabs and the suspensions were enriched in Tryptic Soy broth (ThermoFisher Oxoid, Italy) for 6 h at 37 °C and then cultured onto Columbia Blood Agar, Columbia CNAM Agar, Mannitol Salt Agar, MacConkey Agar, Hektoen Enteric Agar (Liofilchem®, Italy), and Yersinia CIN Agar (ThermoFisher Oxoid, Italy). Selective and chromatic media (Liofilchem®, Italy) were used to detect *Staphylococcus aureus*, methicillin-resistant *S. aureus*, *Pseudomonas aeruginosa*, *Bacillus cereus*, *Salmonella* spp., after pre-enrichment in Rappaport Vassiliadis broth, *Burkholderia cepacia* complex, *Listeria* spp., after pre-enrichment in Half-Frase broth and Frase broth, *Vibrio* spp., *Enterobacter sakazakii*, *Helicobacter pylori*, *Campylobacter* spp., *Clostridium* spp., following standard protocols [68]. Plates were incubated for 24 h at 37 °C aerobically, anaerobically or in microaerophilic atmosphere (CampyGen Oxoid, Italy). In all cases in which bacteria growth was not observed, plates were incubated for a further 24 h before being classified as negative or, in the case of subsequent growth, underwent bacterial identification. Moreover, for each anatomical district, the qualitative and quantitative *Mycoplasma* spp. and *Ureaplasma* spp. detections were carried out using Mycoplasma System Vet (Liofilchem®, Italy). For the detection of yeasts and fungi, Sabouraud Dextrose Agar with chloramphenicol (Liofilchem®, Italy) and Mycosel Agar (Liofilchem®, Italy) plates were incubated at 30 °C aerobically, and examined daily over a 14 d period before being classified as negative.

Bacterial isolates were identified using standard microbiological procedures, as growth and colonial characteristics, Gram staining, cellular morphology, catalase and oxidase reactions, coagulase test (Coagulase Test, Liofilchem®, Italy), and hemolysin production. Species identification was carried out using the biochemical gallery systems (Remel Oxoid, Italy; API bioMérieux, France; Liofilchem®, Italy). Serological identification of *Salmonella* spp. was performed by a slide agglutination method following the Kauffmann-White-Le Minor scheme [69].

Identification of filamentous fungi was achieved at the genus or species level. Yeast colonies were identified by macro- and micro-morphologic characteristics and based on morphological and biochemical characteristics, such as the presence of capsule by India Ink testing, urease production at 25 °C, and the germ tube test. Biochemical identification was performed by Integral System Yeast (Liofilchem®, Italy).

For each plate, the number of colony forming units (CFU), was converted into number of bacteria or fungi or yeast per ml of saline solution, equal to the number of microflora per swab, following standard methods [70].

### Virological examination

Swabs from oral cavity (*n* = 10) and cloaca (*n* = 10) were collected for virological examination. DNA was extracted using the DNeasy kit (Qiagen, Hombrechtikon, Switzerland) following the manufacturer’s instructions. The obtained DNA (assessed for purity and quantified with a spectrophotometer (Nanodrop, Thermofischer, Reinach, Switzerland) was used as a template (100 ng total, per sample) in distinct PCR reaction mix that was prepared and tested to detect the presence of herpesvirus, adenovirus and iridovirus DNA according to established protocols (panherpesvirus, [71]; panadenovirus, [72]; iridovirus, [73]) together with the proper reaction control samples.

### Parasitological examination

Ticks collected from each tortoise were mounted in Hoyer medium and microscopically examined for their identification at species level, by using the taxonomic keys [74–76]. Fecal samples were analyzed macroscopically for helminths (e.g., adult nematodes, proglottids of cestodes, and worm fragments), and then microscopically for helminths and protozoa. For microscopical analysis, we used a flotation test with a low-density solution (saturated NaCl solution, specific gravity 1.2).

In order to evaluate the efficacy of fenbendazole treatment against oxyurids, a fecal egg count reduction (FECR) test was performed on samples prior and about 1 month after deworming with Fenbendazole (75 mg/kg, repeated with 50 mg/kg after 10 days) by using a low density solution (specific gravity 1.2) McMaster technique with a sensitivity of 50 eggs per gram (EPG) [62]. Pre- and post-treatment fecal egg counts (FEC) were used to calculate the reduction of fecal egg counts (FECR) according to the formula FECR = 100 * (1 - FEC post-treatment/FEC pre-treatment); FECR ≥90% indicated the efficacy of the treatment [59].

### Statistical analysis

Frequencies of isolated microorganisms were compared among anatomical districts, sexes and, in the case of bacteria, Gram affinity, using Chi-square (χ^2^) and Fisher’s exact tests. Continuous variables are reported as means ± standard deviations and were analyzed using Student’s *t*-tests or ANOVA followed by Holm-Sidak post-hoc test when indicated. Statistical tests were performed using software STATA version 13 (STATA Corporation, College Station, Texas, US). *P*-values less than 0.05 were considered significant.

## Data Availability

The datasets used and/or analysed during the current study are available from the corresponding author on reasonable request.

## Abbreviations

ANOVA: Analysis of variance
Bcc: *Burkholderia cepacia* complex
C: Degrees celsius
CFU: Colony-forming unit
CIN: Cefsulodin-irgasan-novobiocin
CITES: Convention on international trade of endangered species
CNAM: Colistin and nalidixic acid modified
d: Day
d.f.: Degrees of freedom
DNA: Deoxyribo nucleic acid
EPG: Eggs per gram
e.g.: For example (lat: *Exempli gratia*)
F: Result of ANOVA test
FEC: Fecal egg count
FECR: Fecal egg count reduction
h: Hour
IUCN: International union for conservation of nature
kg: Kilogram
m: Minute
mg: Milligram
ml: Milliliter
mm: Millimeter
μl: Microliter
μm: Micrometer
n: Number
*P*: *P*-value
PCR: Polymerase chain reaction
s: Second
SCUD: Septicemic cutaneous ulcerative disease
STATA: Software for statistics and data science
t: Result of student’s t-test
χ^2^: Chi-square
